# A randomized controlled trial to test the effects of displaying the Nutri-Score in food advertising on consumer perceptions and intentions to purchase and consume

**DOI:** 10.1186/s12966-024-01588-5

**Published:** 2024-04-15

**Authors:** Didier Courbet, Laure Jacquemier, Serge Hercberg, Mathilde Touvier, Barthélémy Sarda, Emmanuelle Kesse-Guyot, Pilar Galan, Nicolas Buttafoghi, Chantal Julia

**Affiliations:** 1 Aix-Marseille Univ, Université de Toulon, IMSIC, InCIAM, Marseille, France; 2 Aix-Marseille Univ, CRETLOG, Aix-en-Provence, France; 3 Center of Research in Epidemiology and StatisticS (CRESS), Nutritional Epidemiology Research Team (EREN), Université Sorbonne Paris Nord and Université Paris Cité, INSERM, INRAE, CNAM, Bobigny, 93017 France; 4 Public Health Department, Avicenne Hospital, Assistance Publique des Hôpitaux de Paris (AP-HP), Bobigny, France; 5 Kedge Business School, Marseille, France; 6 Département Techniques de Commercialisation, Aix-Marseille University, IUT d’Aix-en-Provence, 413 Avenue Gaston Berger, Aix-en-Provence, 13100 France

**Keywords:** Nutri-Score, Nutrition labelling, Advertising messages, Perceived quality product, Public health

## Abstract

**Background:**

Some research shows that advertising for high-fat, sugar, or salt (HFSS) products is contributing to a shift in consumer preferences toward products of poor nutritional quality, leading to unhealthy nutritional intakes that increase the risk of obesity and chronic diseases. A strategy of displaying simple and understandable nutritional information (like the front-of-pack nutrition label Nutri-Score) in food messages could be an aid to help guide consumers’ choice towards healthier products.

**Methods:**

A randomized controlled experiment was conducted on 27,085 participants randomly assigned to two experimental conditions or a control condition. In both experimental conditions (independent variable: advertising messages with *vs.* without the Nutri-Score), participants were exposed to advertisements for diversified food products with contrasting nutritional quality and belonging to nine different food categories. Participants were then asked questions about their perception, affective evaluation, and intentions to purchase and consume the products. In the control condition, they were not exposed to the advertisements.

**Results:**

Overall, interaction effects between the two variables (1) the messages with *vs.* without the Nutri-Score and (2) the nutritional quality of products, were significant for all dependent variables, with effect sizes between large and medium. Overall, the better the products’ nutritional quality, the more positive their perceptions, affective evaluations, and intentions to buy and consume them. When the Nutri-score was displayed in advertising messages (vs. when it was not), perceptions, affective evaluation, and behavioral intentions: (1) became more positive for products of good nutritional quality (Nutri-score A and B), (2) became more negative for products of poor nutritional quality (Nutri-score D and E), (3) changed little or not at all for products of intermediate nutritional quality (Nutri-Score C).

**Conclusions:**

This research is the first in the literature to demonstrate that displaying the Nutri-Score in advertising messages assists consumers in directing their choices towards healthier foods. Regulations mandating the display of the Nutri-Score in food advertising could be an effective public health measure.

**Supplementary Information:**

The online version contains supplementary material available at 10.1186/s12966-024-01588-5.

## Introduction

The current food environment promotes the consumption of processed foods high in nutrients of concern (saturated fat, salt, and sugar) through commercial strategies involving advertising on traditional or digital media [1]. Many studies show how it is difficult for individuals to “resist” the influence of advertising for high-fat, sugar, or salt (HFSS) products. Not only are these products an important source of pleasure [2], but advertisements also use persuasive processes that are often very effective [3]. In the face of such obesogenic environments driven by commercial determinants of health, governments struggle to adopt efficient policies to reduce the burden of non-communicable diseases [4]. While some countries, such as the United Kingdom or Chile, have implemented regulations to limit the deleterious effects of advertising for HFSS products, very limited progress has been made elsewhere towards effectively regulating advertising of this type of products [5, 6], although they are the most prevalent products being advertised [7].

In the absence of more stringent and effective regulations, such as banning advertising for foods of low nutritional quality, a possible strategy is to help people “resist” the influences of advertising messages for HFSS products. Research explains the processes that need to be in place to accomplish this [8]. Overall, the recommendations are for children, adolescents, and adults alike [9]. To resist effectively, individuals must have access to simple, easily understandable information relating to global nutritional quality of foods in order to form cognitive and affective judgements about products (e.g. attitude) and behavioral intentions (e.g. relating to purchases or personal consumption) [8]. These judgments are major determinants of behavior with products (intentions to purchase, to consume, to give to children …) [9].

In everyday life, most of the time, advertising messages are received and processed very quickly by people who pay little or no attention to them. Thus, though they may have nutritional knowledge about the foods, people may not be able to act upon them, because, for example, they are thinking about something else and do not have enough attentional resources available at that moment [9]. In addition, they may not be sufficiently motivated to access the nutritional information in their memory and then to consider it in their judgments and behavioral intentions towards the products. Thus, providing nutritional quality information at the time of receipt would mitigate the deleterious effects of advertising for HFSS products.

Implemented on a voluntary basis in several countries (France, Spain, Belgium, Germany, Luxembourg, the Netherlands, and Switzerland in particular), the Nutri-Score is a color-coded, summary-graded label providing an overall evaluation of the nutritional value of a food or beverage, from A/green (higher nutritional value) to E/red (lower nutritional value). Studies have shown that front-of-pack labeling (FoPL) and, in particular, the Nutri-Score can help consumers identify the nutritional quality of products [10, 11]. It then influences food choices by directing them towards healthier products [12–15]. Thus, as the Nutri-Score can effectively and quickly inform about the overall nutritional quality of foods, displaying it in advertising messages could reduce the deleterious effects of advertising for HFSS products. In France, where this study was conducted, there are no legal requirement on the use of nutritional labeling in food advertising messages. There are also no legal restrictions to display it, and enforcing its implementation on a mandatory basis could be a policy option.

However, to the best of our knowledge, no study has investigated the effects of displaying the Nutri-Score in advertising messages on consumer perceptions, evaluations, and behavioral intentions. Thus, the objective of the present study was to evaluate the effects of the Nutri-Score in food advertising messages on the main determinants of behavior, i.e., cognitively, affectively, and in terms of behavioral intentions, in adult participants.

## Methods

### Research design

**Fig. 1:**
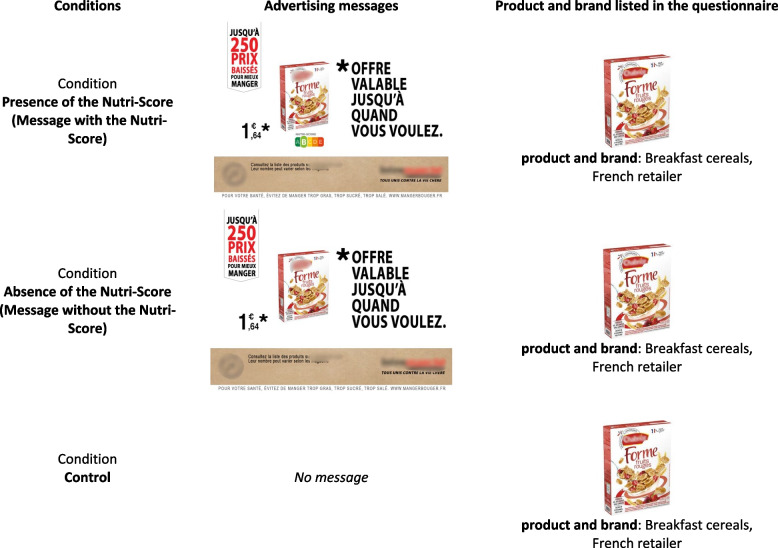
Examples of advertising messages and products used in experimental and control conditions. Translation of advertising messages: *Offer valid until whenever you want—Up to 250 lower prices to eat better. All united against high prices

The study design consisted of three conditions into which participants were randomly assigned: exposure to advertising messages with the Nutri-Score displayed *vs.* exposure to advertising messages without the Nutri-Score *vs.* no exposure to advertising messages (control condition) (Fig. 1). The independent variable was called the “messages with *vs.* without the Nutri-Score” variable.

### Participants

Participants were recruited from the NutriNet-Santé cohort study. Briefly, the NutriNet-Santé cohort study is a web-based cohort study aimed at understanding the determinants of dietary behavior and the relationship between nutrition and health [16]. Volunteers aged 18 or older were recruited through various multimedia channels (https://etude-nutrinet-sante.fr/). All participants were required to provide electronic informed consent when enrolling in the study. The NutriNet-Santé study is conducted according to the Declaration of Helsinki guidelines and is registered at clinicaltrials.gov (NCT03335644). The study was approved by the Institutional Review Board of the French Institute for Health and Medical Research 47 (IRB Inserm n°0000388FWA00005831) and the “Commission Nationale de l’Informatique et des 48 Libertés” (CNIL n°908450/n°909216).

After completing a series of questionnaires, including those related to their socio-demographic characteristics, participants in the cohort were regularly invited to participate in protocols investigating specific areas of research on dietary behavior or health. Between January 2020 and March 2020, participants were invited to participate in a study investigating the perception of products sold on the market. A total of 27,085 participants took part in this specific study. The demographic profile of the participants is presented in Table 1.

**Table 1:** Demographic data of the sample (*n* = 27,085)

Number	*n* = 27,085
**Gender, n (%)**	
Male	7,155 (26)
Female	19,930 (74)
**Age, n (%)**	
Under 19 years old	36 (0.1)
20–29 years old	919 (3)
30–39 years old	2,878 (11)
40–49 years old	3,858 (14)
50–59 years old	5,487 (20)
60–69 years old	7,854 (29)
70–79 years old	5,423 (20)
80 years old and older	630 (2)
**Body mass index (BMI), Mean (SD)**	24 (4)
**Socio-professional class, n (%)**	
Farmers	142 (1)
Craftsmen, merchants, company managers	1,147 (4)
Executive and higher intellectual professions	11,383 (42)
Intermediate professions	6,881 (25)
Office workers	6,426 (24)
Laborers	424 (2)
Retired	134 (0.5)
Students	133 (0.5)
Non-working	113 (0.5)
Missing	302 (1)
**Education Level, n (%)**	
High school diploma or less	4,604 (17)
Bachelor’s degree	3,451 (13)
University degree or higher	18,593 (69)
Missing	437 (2)
**Income, n (%)**	
Less than 1,430 €/month OR less than 17,170 €/year	1,553 (6)
from 1,430 to 2,000 €/month OR from 17,170 to 24,050 €/year	2,374 (9)
from 2,000 to 2,700 €/month OR from 24,050 to 32,290 €/year	3,644 (13)
from 2,700 to 3,780 €/month OR from 32,290 to 45,400 €/year	5,688 (21)
from 3,780 to 4,800 €/month OR from 45,400 to 57,550 €/year	4,310 (16)
from 4,800 to 8,710 €/month OR from 57,550 to 104,550 €/year	4,435 (16)
More than 8 710 €/month OR more than 104 550 €/year	753 (3)
I do not know	622 (2)
I do not wish to answer	3,414 (13)
Missing	292 (1)

### Materials

#### Products

In order to increase the external validity of the study, 39 food products with a Nutri-Score ranging from A to E were selected to provide a large number of different food products, that were also diversified on the basis of several criteria. They belonged to 9 different food groups: cereals, beverages, breakfast, bars, cookies, salty snacks, cold cuts, ready meals, and desserts (see Additional file 1). Several reasons motivated these choices: 1) the different food groups were related to various consumption moments (breakfasts, snacks, aperitifs, meals) associated with potentially different customer segments (children, adults); 2) these groups gave the possibility, on the one hand, of having, in total, products of very contrasting nutritional quality, ranging from “higher nutritional quality” (Nutri-Score A) to “lower nutritional quality” (Nutri-Score E). On the other hand, they made it possible to vary, within the same group, the nutritional quality of the products, reflected by at least three different Nutri-Score levels; 3) marketed for several years in France, they were sold in small, medium, and large food stores at the time of the study.

#### Advertising messages

Each of the 39 products was integrated into fixed advertising messages that a graphic designer fashioned for the experiment and in which the Nutri-Score was not included. From the 39 ads, a second series of 39 ads was designed by simply displaying the appropriate Nutri-Score for each marketed product. The Nutri-Score was placed next to the product in close-up. The choice of the surface area occupied by the Nutri-Score label on each message was made according to the ratio: surface area of the Nutri-Score logo/total surface area of the print message, as observed in print advertisements distributed in France and in which the Nutri-Score is displayed. The ratio was based on an exploratory study, which showed that in the latter messages, this ratio varies from 1% (Nesquik cereals) to 2% (Roast Pork Auchan). We opted for the lowest ratio (1%) so as not to present a Nutri-Score logo that would attract too much attention from the participants and then risk reinforcing the possible experimental effects. This ratio was identical in the 39 designed messages. Thus, there were 39 pairs of nearly identical messages. The only difference between each pair was the presence or absence of the Nutri-Score.

### Procedures

The survey was administrated online. Participants were informed that this was a scientific study with no commercial connection to the brands presented. In both experimental conditions, it was stated that the objective of the survey was to evaluate the effects of advertising messages for different food products. Participants were asked to watch the messages as if they were watching them in everyday life and then to answer questions about the products on display. Participants were then exposed to six blocks, each block consisting of an advertisement for one product followed by six questions about the product.

In total, each participant saw six advertisements, each for a different product. Each of the six products belonged to a different food category. The choice of products was made in the following way: a computer program randomly selected six food groups from the nine available groups. Within each group, the computer randomly selected a product, along with its advertisement. The choice was made to evaluate 6 products (among the 9) in order to limit the administration time of the questionnaire (average administration time measured during the pre-test = 9 min) and to avoid participant fatigue.

In the control condition, participants did not see the advertisements but only the food products alone. Under the same conditions as for participants in the experimental groups, they answered six questions about six products randomly selected according to the same rules as indicated above. Each product was represented by a photo with the name under which it was marketed (Fig. 1). In all three experimental and control conditions, it was made clear that there were no right or wrong answers: only the participants’ impressions mattered.

In each condition, a pre-test of all the procedures was carried out on 14 people of various ages and socio-demographic profiles. This made it possible to ensure that the objectives and questions were well understood, that the material was easy to read and that, at each stage, the experiment conditions were well implemented.

### Measurements

Measures were based on research in the areas of (1) the effects of persuasive communication, specifically attitude change through messages, including advertising [17, 18], and (2) the determinants of nutritional behavior: (1) cognitive and affective determinants, (2) behavioral intentions [19].

The first two measures were cognitive and pertained to

1. the perceived nutritional quality of the product (scale from 1: very bad to 7: very good);
2. the perceived healthiness of the product (scale from 1: very unhealthy to 7: very healthy);
3. the third measure is affective and concerns the affective evaluation of the product, i.e., the attitude towards the product (scale from 1: very unfavorable to 7: very favorable).

The other measures concerned behavioral intentions. They concerned three types of diversified actions linked to different targets with (4) intention to purchase the product (scale from 1: not at all to 7: completely) and (5) intention to consume the product personally with scales of quantities/frequency of consumption, indicating the quantity per month, per week, or per day (see Additional file 1); (6) intention to give the product to a child aged 7 to 12 years with scales of quantities/frequency of consumption indicating the quantity per month, per week, or per day. If each scale differed according to the type of product, there was a systematic increasing order of graduation that went from 1: never to 7: high quantities/frequencies.

For the same participant, the order of the six questions was randomized each time. The polarity of the scales was also randomized between participants: the number one (*vs.* 7) is sometimes on the left, sometimes on the right of the scale.

### Data analysis

The analyses were conducted with Statistica version 13 (StatSoft). To determine the factor structure of the results, we performed a principal component analysis (PCA) on all the dependent variables (Additional file 2). For the number of factors to be retained, we used the Empirical Kaiser Criterion method (eigenvalues > 1) [20] and the Scree-test based on the eigenvalue curve [21]. To analyze the effects of the “messages with *vs.* without the Nutri-Score” variable in relation to the Nutri-Score, we performed variances analyses (ANOVA) to analyze the main effects and interactions. The effect size is calculated with ηp^2^. Small, medium, and large effect sizes correspond to a value of ηp^2^ = 0.01; 0.06; and 0.14, respectively [22]. To better understand the effects of the variables involved, we compared the different effect sizes [23]. The Student’s t-test was used to compare two means. Effect size was calculated with Cohen’s d. Small, medium, and large effect sizes corresponded to a value of d = 0.2; 0.5; and 0.8, respectively [22]. For all analyses, *p* < 0.05 was considered statistically significant.

## Results

### Overall results

In the Principal Component analysis of all six dependent variables (Additional file 2), a general factor was retained. It satisfactorily explained 71.5% of the total variance (Table 2).

**Table 2:** Factor analysis (principal component analysis) based on the six dependent variables

Dependent variables	General factor
Perceived product nutritional quality	**0.84**
Perceived healthiness of the product	**0.85**
Affective evaluation of the product (attitude)	**0.91**
Intention to purchase the product	**0.81**
Intention to personally consume the product	**0.82**
Intention to give the product to a child between 7 and 12 years old	**0.83**
Eigenvalues	4.29
% total explained variance	71.5

**Fig. 2:**
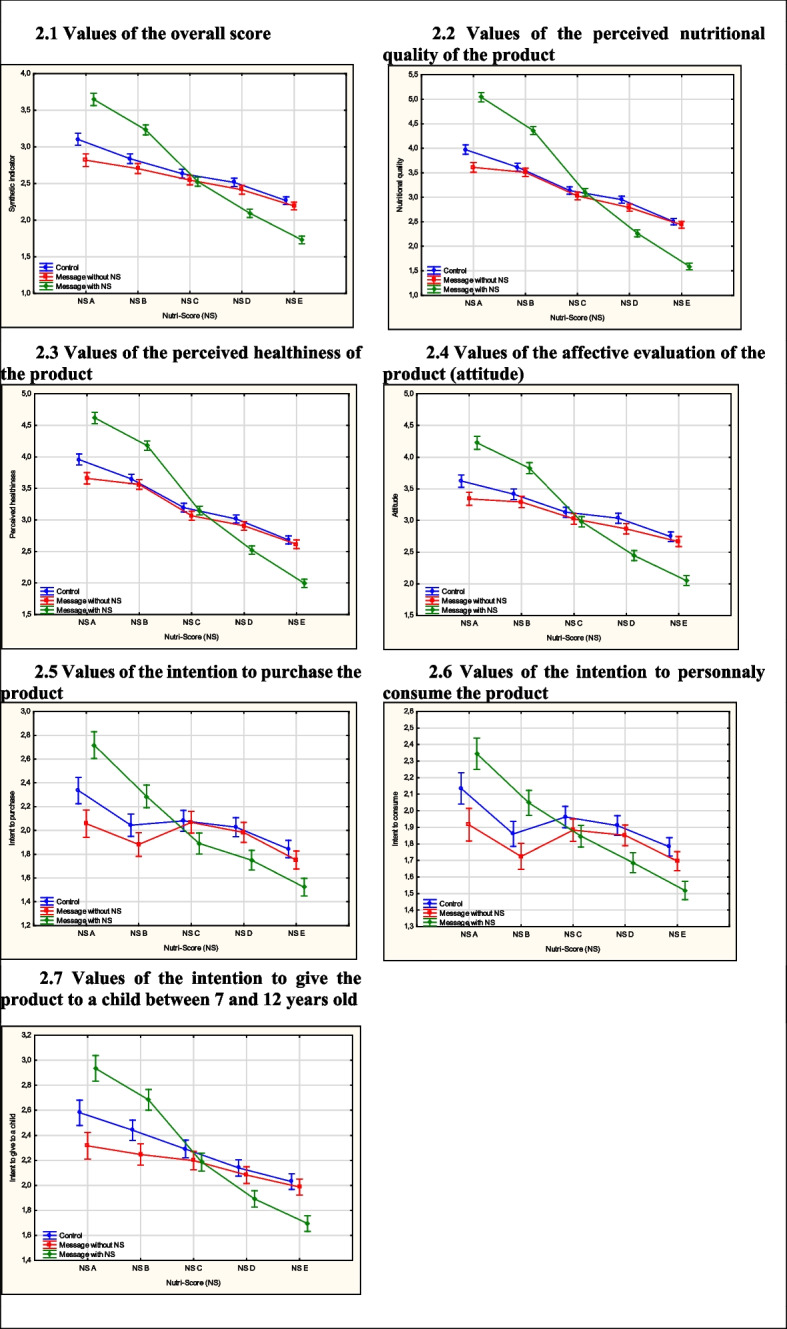
Values of the seven dependent variables according to the “messages with *vs.* without the Nutri-Score *vs*. no message” variable and the nutritional quality of the products (Nutri-Score from A to E). Notes. The vertical bars represent the confidence interval at .95. NS means Nutri-Score

The six dependent variables were therefore aggregated into a single overall score, which represented their average. This one was calculated after testing for the homogeneity of variances and normality of distributions of the aggregated variables. This indicator summarizes the overall effects. The ANOVA used the following design: 3 (messages with *vs.* without the Nutri-Score *vs.* no message) × 5 (nutritional quality of the products: Nutri-Score A; Nutri-Score B; Nutri-Score C; Nutri-Score D; Nutri-Score E). This first of all revealed the main significant effects of the “messages with *vs.* without the Nutri-Score *vs.* no message” variable: F (22798) = 10.29, *p* < 0.001, η_p_^2^ = 0.007; of the “nutritional quality of the products” variable: F (411192) = 777.06, *p* < 0.001, η_p_^2^ = 0.217. Secondly, interaction effects were identified: “messages with *vs.* without the Nutri-Score *vs.* no message” variable × “nutritional quality of products” variable: F (811192) = 116.33, *p* < 0.001, η_p_^2^ = 0.077 (Fig. 2).

To analyze the size of the effects due to the display of the Nutri-Score in the advertising messages, we compared the scores of the dependent variables according to the presence or absence of the Nutri-Score in the commercials, in relation with the nutritional quality of the product (Table 3).

**Table 3:** Differences in the effects of messages with the Nutri-Score *vs.* without the Nutri-Score on the seven dependent variables, in relation with the nutritional quality of the products: analysis of variance and effect size (η_p_^2^)

	Overall Score	Perceived product nutritional quality	Perceived healthiness of the product	Affective evaluation of the product	Intention to purchase the product	Intention to personally consume the product	Intention to give the product to a child
	η_p_^2^	η_p_^2^	η_p_^2^	η_p_^2^	η_p_^2^	η_p_^2^	η_p_^2^
	F (df)	F (df)	F (df)	F (df)	F (df)	F (df)	F (df)
Source of effects:							
Messages with *vs.* without the Nutri-Score	.007	.017	.008	.001	.002	.003	.005
	12.32 (1.1826)^***^	30.84 (1.1826)^****^	14.48 (1.1826)^***^	2.71 (1.1826)	3.84 (1.1826)^*^	4.95 (1.1826)^*^	9.10 (1.1826)^**^
Nutritional quality	.276	.454	.386	.223	.05	.038	.09
	696.96 (4.7304)^****^	1518.27 (4.7304)^****^	1148.39 (4.7304)^****^	523.15 (4.7304)^****^	96.95 (4.7304)^****^	72.43 (4.7304)^****^	179.65 (4.7304)^****^
Messages with *vs.* without the Nutri-Score ^*^Nutritional quality	.099	.168	.104	.074	.03	.02	.035
	200.04 (4.7304)^****^	369.51 (4.7304)^****^	211.93 (4.7304)^****^	146.59 (4.7304)^****^	55.34 (4.7304)^****^	40.04 (4.7304)^****^	67.03 (4.7304)^****^

η_p_^2^ = Eta^2^; F = ANOVA F-value; (df) = degrees of freedom

^*^p < 0.05

^**^p < 0.01

^***^p < 0.001

^****^p < 0.000001

ANOVAs revealed significant interaction effects of the two variables “messages with *vs.* without the Nutri-Score” and “nutritional quality of the products” on all the dependent variables (*p* < 0.000001). The size of these interaction effects on the “overall score” variable was between “large” to “medium” (ηp^2^ = 0.099). Generally, the effects on the overall score were representative of those observed on the other dependent variables. Indeed, in the ‘messages without the Nutri-Score’ condition, the results showed that the values of the overall score decreased as the nutritional quality of the products decreased. In the ‘messages with the Nutri-Score’ condition, the values of the overall score also decreased as the nutritional quality of the products decreased. However, the difference between the two randomization arms was more pronounced at both ends of the nutritional quality scale, similar to an “amplification effect”. Thus, the differences in values were higher (1) for products with underlying Nutri-Scores A and B, with significantly higher values for the ‘messages with the Nutri-Score’ condition and (2) for products with Nutri-Scores D and E, with significantly lower values for the ‘message with the Nutri-Score’ condition. The largest significant difference concerned products with Nutri-Score A: m Nutri-Score = 3.65 *vs.* m without Nutri-Score = 2.82, t (10824) = 31.9, *p* < 0.001, d = 0.67. Note that the judgments about products with Nutri-Score C were not affected by the display of the latter in the messages: m Nutri-Score = 2.52 *vs.* m without Nutri-Score = 2.54, t (15647) = 0.86, p = 0.39.

**Fig. 3:**
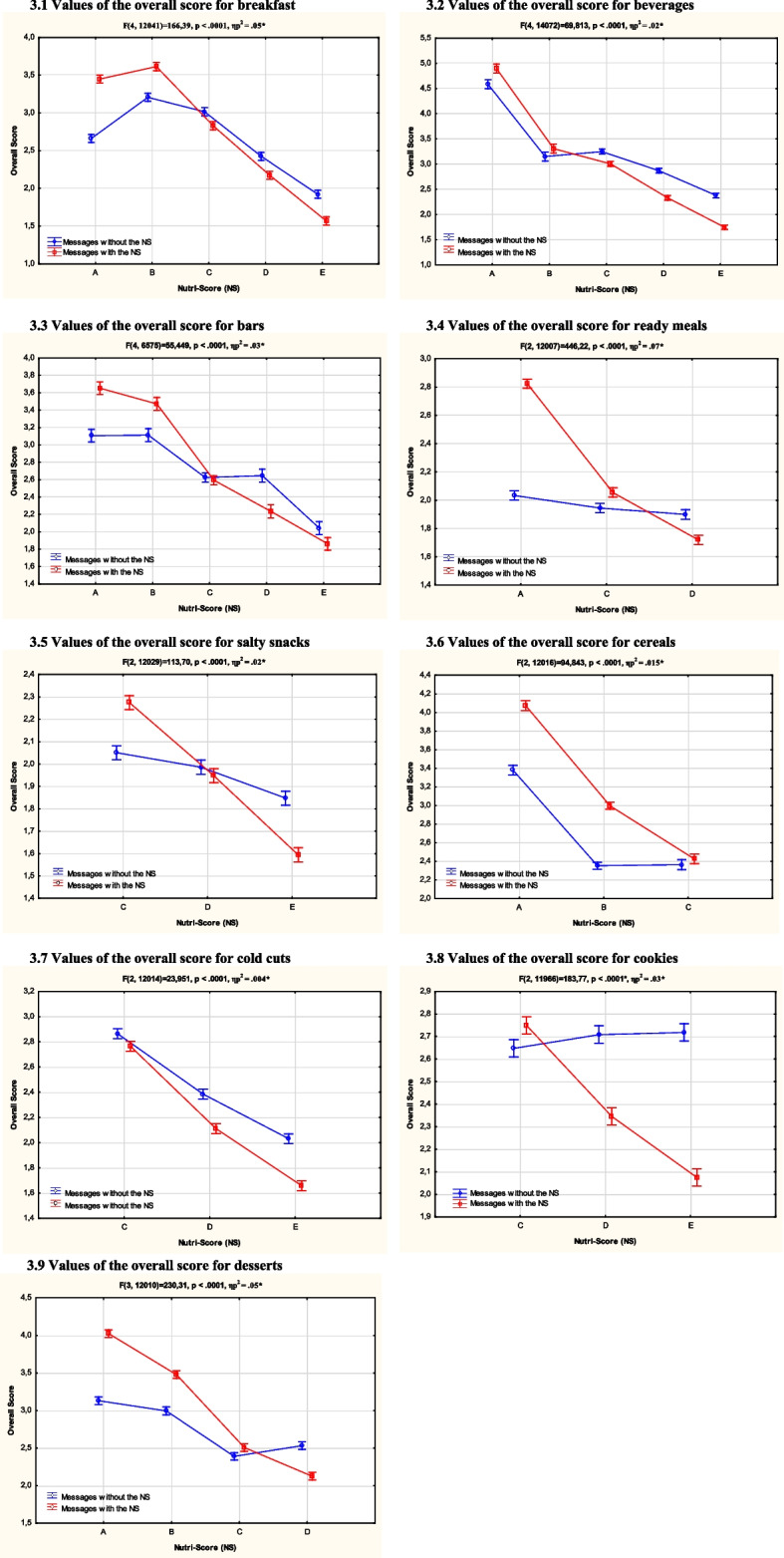
Values of the overall score for 9 food categories according to the “messages with vs. without the Nutri-Score” variable and the nutritional quality of the products (Nutri-Score from A to E). Notes. The vertical bars represent the confidence interval at .95. NS means Nutri-Score. * Results of the ANOVA using the following design: 2 (messages with vs. without the Nutri-Score) × 5 (nutritional quality of the products: Nutri-Scores A, B, C, D, E)

It should be noted that the food product group had a very limited – though statistically significant—effect size with the two variables above. Indeed, the ANOVA used the following design: 2 (messages with *vs.* without the Nutri-Score)) X 9 (food category: cookies, cereals, breakfast, ready meals, salty snacks, drinks, bars, desserts, deli) revealed an effect size of η_p_^2^ = 0.001 (F (2107968) = 62.83, *p* < 0.001). Similarly, the ANOVA used the following design: 2 (messages with *vs.* without the Nutri-Score) × 5 (nutritional quality of the products: Nutri-Score A; Nutri-Score B; Nutri-Score C; Nutri-Score D; Nutri-Score E) X 9 (food category: cookies, cereals, breakfast, ready meals, salty snacks, drinks, bars, desserts, deli) revealed an effect size of η_p_^2^ = 0.002 (F (21107968) = 10.59, *p* < 0.001). Due to the very low values of these two effect sizes [22], they were considered negligible. Results by food category are provided in Additional file 3 and Fig. 3.

Furthermore, it is observed that the values of the overall score are lower in the condition of messages without Nutri-Score compared to the control condition, with a small effect size. An ANOVA was conducted to examine the main effects and interaction effects within the experimental design involving 2 (messages without the Nutri-Score vs. no message) × 5 (nutritional quality of the products: Nutri-Score A; Nutri-Score B; Nutri-Score C; Nutri-Score D; Nutri-Score E). This analysis initially revealed significant main effects associated with (a) the “messages without the Nutri-Score vs. no message” variable (m = 2.5 and m = 2.6, respectively): F (1,1858) = 17.6, *p* < 0.001, η_p_^2^ = 0.009; (b) the “nutritional quality of the product” variable: F (4,7432) = 196.9, *p* < 0.001, η_p_^2^ = 0.09. Subsequently, interaction effects were observed: messages without the Nutri-Score vs. no message × nutritional quality of products: F (47432) = 4.64, *p* = 0.001, η_p_^2^ = 0.002 (Fig. 2). The very small size of these interaction effects may be considered negligible.

### Specific results concerning the different dependent variables

The interaction effects size of the two variables “messages with *vs.* without the Nutri-Score” and “nutritional quality of the products” varied according to the type of dependent variable. The results are presented below from largest to smallest interaction effect. The largest interaction effect sizes were for the two cognitive variables. First, for the dependent variable “perceived nutritional quality of the product”, the effect size ηp^2^ = 0.168 was between “very large” and “large” [22]. The most important effects concerned products with Nutri-Score A: m Nutri-Score A = 5.03 *vs.* m without Nutri-Score A = 3.72, t (10824) = 47.4, *p* < 0.001, d = 0.91. Then products with Nutri-Score E: m Nutri-Score = 1.64 *vs.* m without Nutri-Score = 2.44, t (13543) = 48, *p* < 0.001, d = 0.82. We note that the perception of the nutritional quality of products with Nutri-Score C was only slightly modified by the display of the latter: m Nutri-Score = 3.18 *vs.* m without Nutri-Score = 3.06, t (15647) = 7.32, *p* < 0.001, d = 0.11.

Second, for the ‘healthy product’ variable, the effect size ηp^2^ = 0.104 was between “large” and “medium”. Third was the size of the interaction effects on the “affective evaluation of the product” variable ηp^2^ = 0.074, which was between “large” and “medium”. With an effect size between “medium” and “small”, the effects on the three behavioral variables came next. First “the intention to give to a child aged 7–12” ηp^2^ = 0.035, then “the intention to purchase the product” ηp^2^ = 0.03 and “the intention to personally consume the product» ηp^2^ = 0.02.

## Discussion

Our results show the existence of interaction effects between the “messages with *vs.* without the Nutri-Score” variable and the global nutritional quality of the products. Overall, all dependent variables considered, the size of these interaction effects is between “large” and “medium”. The display of the Nutri-Score in advertising messages (*vs.* the absence of the Nutri-Score) led to a significant "amplification” of (1) cognitive and affective judgements and (2) behavioral intentions in relation to foods with the Nutri-Score above and below the median rating C. The most positive judgments, in the “messages without the Nutri-Score” condition (products with Nutri-Score A and B), became even more positive when the Nutri-Score was displayed. The most negative judgments in the “messages without the Nutri-Score” condition (products with Nutri-Score D and E) became even more negative when the Nutri-Score was displayed. Judgments concerning products with Nutri-Score C, i.e., median scores, were modified only very slightly or not at all by the display of the Nutri-Score in the messages.

Among the five Nutri-Score values, the strongest changes in affective evaluations, in perceptions of the products’ nutritional quality and health benefits, as well as in intentions to purchase, consume and give them to a child between 7 and 12 years old, were observed for products with a Nutri-Score of A. To the best of our knowledge, this research is the first in the literature to show that the display of the Nutri-Score in advertising messages firstly results in a “deterioration” of (1) cognitive and affective judgments and (2) behavioral intentions regarding advertised products of poor nutritional quality. Secondly, it improves (1) cognitive and affective judgments and (2) behavioral intentions regarding advertised products of higher nutritional quality. The Nutri-Score assists consumers in directing their choices towards healthier foods.

The contrasting effects obtained for the different Nutri-Scores show that they cannot be explained by a single health halo effect where, for example, the mere presence of a nutritional rating system, independently of the ratings themselves, would have favorably modified the judgments about the products [24]. Nor can the results be explained by a single two-part categorization effect [25] in which, on the one hand, there would be an improvement in judgments for products perceived as “healthy,” and on the other hand, a “deterioration” in judgments for products perceived as “unhealthy.” In fact, the effects of Nutri-Score C in the messages are specific to this score. The results are consistent with previous research on resistance to the influences of HFSS food advertising [8, 9]. Indeed, works explain that receivers can implement several types of processing of advertising messages. Thus, participants would have allocated enough cognitive resources to consider both the information present in the advertising message and the nutritional information displayed by the Nutri-Score in their cognitive and affective judgments, as well as in their behavioral intentions. Based on the size of the interaction effects between the messages with *vs.* without the Nutri-Score and the nutritional quality of the products, we know more precisely on which dependent variables the “amplification effects” of the judgments were the most important. The size of the most important interaction effects concerned the two cognitive judgments: first, the perceived nutritional quality of the product, where the effect size was judged to be between “very large” and “large”, and second, the perceived healthiness of the product, where the effect size was between “large” and “medium”.

The level of nutritional quality indicated by the Nutri-Score in the advertisements was well perceived, understood, and incorporated into both cognitive judgments about the products. In addition, participants effectively linked the nutritional quality of the products indicated by the Nutri-Score to health. The presence of the Nutri-Score in the advertisements produced significant effects in making relevant judgements about the nutritional quality of the advertised product and whether it is “more or less favorable” to health. These two cognitive judgments are important for several reasons. They are evidence that the nutritional information has been considered. In addition, this type of information can then be disconnected from the advertising messages, easily remembered, passed on, and taught to others, especially children [26].

Previous studies have shown that displaying the Nutri-Score on product packaging allows (1) better knowledge of the global nutritional quality of foods and healthier food choices [10–15] and (2) to limit the potential halo effects on health of nutritional claims about added sugars promoted by companies on packaging (for example, 30% less sugar) which may make products appear healthier than they really are [27]. The present study extends these results by showing the existence of effects of the Nutri-Score on cognitive judgments related to perceptions of nutritional quality and more or less favorable health-promoting effects of food products, in the context of advertising.

Regarding the size of the interaction effects between the messages with *vs.* without the Nutri-Score and nutritional quality of the products, the third largest effect concerned the variable “affective evaluation of the product” (attitude toward the product), which was considered to be between “large” and “medium”. This result is important because attitude toward the product is an important determinant of behavior [28]. In addition, as many advertising messages for HFSS products use processes to increase affective evaluations of advertised products and brands [9], the Nutri-Score could limit such affective effects.

The interaction effects on the three behavioral intentions variables come next (effect size between “medium” and “low”). First, the “intention to give to a child aged 7 to 12,” then the “intention to purchase the product,” and finally, the “intention to personally consume the product.” The display of the Nutri-Score in advertisements therefore has the capacity to significantly modify behavioral intentions, which are strongly predictive of the behavior in many studies [28]. Consistent with literature, the effects on behavioral intentions are logically less strong than those on cognitive and affective judgments. Indeed, behavioral intentions depend on multiple other factors such as taste, anticipation of pleasure in eating [2], and psychosocial determinants such as subjective norms, i.e., the social pressure perceived by the individual regarding the consumption of the food (e.g., the perception of household members’ opinions about food [28]). Of the three types of behavioral intentions measured, the most important (*vs.* the least important) effects of the Nutri-Score in the messages are logically related to the act of giving the products to children (*vs.* its personal consumption). Indeed, guaranteeing the good health of children is essential for those who feed them [29].

On an interventional level and regarding the implications of our results for public health policies, this research shows that displaying the Nutri-Score of products in advertising messages has a double advantage: this helps consumers direct their choices towards foods of better nutritional quality, firstly by “moving consumers away” from products of poor nutritional quality and, secondly, by “bringing them closer” to products of good nutritional quality. Thus, changing the regulations in this direction would benefit public health. As it has been shown that diets with – on average – a higher proportion of products of higher nutritional quality according to the Nutri-Score algorithm were associated with reduced risks of non-communicable diseases, this study suggests that displaying the Nutri-Score on advertising may contribute to reducing the burden of non-communicable diseases in the population [30].

Further research is needed to understand why the values of the overall score were slightly lower in the "messages without Nutri-score" condition than in the control condition. The effects of food advertising on children have been more clearly demonstrated than on adults. Less research has been done on adults [31, 32]. Thus, several explanatory hypotheses can be considered. Firstly, the messages consisted of still images and were not associated with sound, whereas when some research has managed to show effects of food brand advertising in adults, it has mainly used moving images with sound (e.g. TV advertising; [32]). Research has shown that the persuasive effects of audiovisual advertising messages differ from those of still advertising messages [33]. Furthermore, the messages used essentially developed “informational” arguments such as price, showing, in addition, a photo of the packaging. The content was therefore not based on playful narratives and characters that encourage positive affective reactions in receivers. Yet the latter are important determinants of the effects of food advertising, including among adults [34]. Since advertisements are abundant in the everyday environment, the six messages seen may have irritated the receivers. From then on, their responses may have been based on the discomfort caused [35].

Secondly, participants may also have implemented processes of resistance to advertising. These processes may have lessened the effects of messages for brands [36]. New studies should be conducted, in particular to develop methodologies for studying cognitions and thoughts at the time of message reception [37].

The experiment also has limitations. The results were obtained from a population that was particularly well informed about the Nutri-Score and volunteered to help researchers advance the science of nutrition. Few respondents were under 24 years of age and the majority of participants were women. However, firstly, analyses of variance were carried out to study all the interaction effects of gender with the two variables (“messages with *vs.*without the Nutri-Score” and “nutritional quality of the product”) on the overall score. The analyses showed no statistically significant results (*ps* > 0.33). Secondly, the majority of household purchases are made by women. Concerning the item “intention to give the product to a child aged 7 to 12 years”: caution should be exercised when interpreting the results because, on the one hand, not all participants are parents of children of this age. On the other hand, the question does not measure the child’s actual behavior. Effects should also be limited to still advertising images. While some products may have displayed nutrition claims, the size of the image in the display during the experiment was not sufficient to allow for an investigation of the interaction between Nutri-Score and nutritional claims. Future studies could be devised to investigate whether the Nutri-Score can act as a moderating factor in the effects of nutritional claims in advertising as it was found on pack [27].

In terms of new research perspectives, the effects of displaying the Nutri-Score in audiovisual advertisements, such as on television or the Internet, and in other commercial communication media outlets, such as in-store advertising and sponsorship, should be tested, just like messages with attractive stories and characters that generate positive emotions. Similarly, the effects on children and adolescents should also be evaluated. Further research is needed to better understand the effects on actual purchasing behavior as well as the longer-term effects, especially when advertising messages are repeated. In terms of the processes involved, a better understanding of the role of the Nutri-Score in processing the advertising messages in which it is included is needed, considering the level of involvement and familiarity with the brand.

## Conclusion

With a randomized controlled experiment carried out on 27,085 individuals, this study is the first in the literature to demonstrate that the display of the Nutri-Score in advertising messages firstly results in a “deterioration” of (1) cognitive and affective judgments and (2) behavioral intentions regarding advertised products of poor nutritional quality. Secondly, it improves (1) cognitive and affective judgments and (2) behavioral intentions regarding advertised products of higher nutritional quality. The Nutri-Score assists consumers in directing their choices towards healthier foods. The implications for public policies related to the prevention of overweight and obesity are significant. In addition to regulating advertising for highly nutritionally unfavorable products to protect children’s health, such as banning advertising from 7 a.m. to 10 p.m. for Nutri-score D and E products [7], mandatory display of the Nutri-score could be an effective measure for adults.

### Supplementary Information

**Additional file 1.** Material: products, advertisements, and quantity-frequency scales used in the experiment.

**Additional file 2.** Results: Descriptive statistics (all food categories considered).

**Additional file 3.** Descriptive statistics: Values of the overall score for 9 food categories according to the “messages with *vs.* without the Nutri-Score” variable and the nutritional quality of the products (Nutri-Score from A to E).

## Data Availability

Researchers from public institutions can submit a collaboration request including information on the institution and a brief description of the project to collaboration@etude-nutrinet-sante.fr. All requests will be reviewed by the steering committee of the NutriNet-Santé study. If the collaboration is accepted, a data access agreement will be necessary, and appropriate authorizations from the competent administrative authorities may be needed. In accordance with existing regulations, no personal data will be accessible.

## Abbreviations

FoPL: Front-of-pack labeling
HFSS food: High-fat, -sugar, or -salt food

## References

[1] World Health Organization Regional Office for Europe. WHO European Regional Obesity: Report 2022. Copenhagen: World Health Organization. Regional Office for Europe; 2022. https://iris.who.int/handle/10665/353747. Licence: CC BY-NC-SA 3.0 IGO.

[2] Stroebe W, Papies EK, Aarts H (2008). From homeostatic to hedonic theories of eating: self-regulatory failure in food-rich environments. Appl Psychol.

[3] Courbet D, Fourquet-Courbet MP. Non-conscious effects of marketing communication and implicit attitude change: state of research and new perspectives. Int J Journalism Mass Comm. 2014;1(1):103.

[4] Mialon M. An overview of the commercial determinants of health. Global Health. 2020;16(1):74. 10.1186/s12992-020-00607-x. 10.1186/s12992-020-00607-x PMC7433173 32807183

[5] Chambers SA, Freeman R, Anderson AS, MacGillivray S (2015). Reducing the volume, exposure and negative impacts of advertising for foods high in fat, sugar and salt to children: a systematic review of the evidence from statutory and self-regulatory actions and educational measures. Prev Med.

[6] Landwehr SC, Hartmann M (2020). Industry self-regulation of food advertisement to children: compliance versus effectiveness of the EU pledge. Food Policy.

[7] Escalon H, Courbet D, Julia C, Srour B, Hercberg S, Serry AJ (2021). Exposure of French children and adolescents to advertising for foods high in fat, sugar or salt. Nutrients.

[8] Hudders L, De Pauw P, Cauberghe V, Panic K, Zarouali B, Rozendaal E (2017). Shedding new light on how advertising literacy can affect children’s processing of embedded advertising formats: a future research agenda. J Advert.

[9] Harris JL, Brownell KD, Bargh JA (2009). The food marketing defense model: integrating psychological research to protect youth and inform public policy. Soc Issues Policy Rev.

[10] Ducrot P, Méjean C, Julia C, Kesse-Guyot E, Touvier M, Fezeu L (2015). Effectiveness of front-of-pack nutrition labels in french adults: results from the NutriNet-Santé cohort study. Gillison F, editor. PLoS One.

[11] Egnell M, Talati Z, Hercberg S, Pettigrew S, Julia C (2018). Objective understanding of front-of-package nutrition labels: an international comparative experimental study across 12 countries. Nutrients.

[12] Dubois P, Albuquerque P, Allais O, Bonnet C, Bertail P, Combris P (2021). Effects of front-of-pack labels on the nutritional quality of supermarket food purchases: evidence from a large-scale randomized controlled trial. J Acad Mark Sci.

[13] Crosetto P, Lacroix A, Muller L, Ruffieux B (2019). Nutritional and economic impact of five alternative front-of-pack nutritional labels: experimental evidence. Eur Rev Agric Econ.

[14] Egnell M, Boutron I, Péneau S, Ducrot P, Touvier M, Galan P (2022). Impact of the Nutri-Score front-of-pack nutrition label on purchasing intentions of individuals with chronic diseases: results of a randomised trial. BMJ Open.

[15] Gassler B, Faesel CK, Moeser A (2023). Toward a differentiated understanding of the effect of Nutri-Score nutrition labeling on healthier food choices. Agribusiness.

[16] Hercberg S, Castetbon K, Czernichow S, Malon A, Mejean C, Kesse E (2010). The Nutrinet-Santé Study: a web-based prospective study on the relationship between nutrition and health and determinants of dietary patterns and nutritional status. BMC Public Health.

[17] Petty RE, Cacioppo JT (1986). The elaboration likelihood model of persuasion. Communication and persuasion.

[18] Zanna MP, Rempel JK, Bar-Tal D, Kruglanski AW (1988). Attitudes: a new look at an old concept. The social psychology of knowledge.

[19] Raine KD (2005). Determinants of healthy eating in Canada: an overview and synthesis. Can J Public Health.

[20] Kaiser HF (1960). The application of electronic computers to factor analysis. Educ Psychol Meas..

[21] Cattell RB (1966). The scree test for the number of factors. Multivar Behav Res.

[22] Cohen J (1988). Statistical power analysis for the behavioral sciences.

[23] Lakens D (2013). Calculating and reporting effect sizes to facilitate cumulative science: a practical primer for t-tests and ANOVAs. Front Psychol.

[24] Chandon P, Wansink B (2007). The biasing health halos of fast-food restaurant health claims: lower calorie estimates and higher side-dish consumption intentions. J Consum Res.

[25] Rozin P, Ashmore M, Markwith M (1996). Lay American conceptions of nutrition: dose insensitivity, categorical thinking, contagion, and the monotonic mind. Health Psychol.

[26] Cullen T, Hatch J, Martin W, Higgins JW, Sheppard R (2015). Food literacy: definition and framework for action. Can J Diet Pract Res.

[27] Jürkenbeck K, Mehlhose C, Zühlsdorf A (2022). The influence of the Nutri-Score on the perceived healthiness of foods labelled with a nutrition claim of sugar. Louie J, editor. PLoS One.

[28] Ajzen I (1991). The theory of planned behavior. Organ Behav Hum Decis Process.

[29] Carnell S, Cooke L, Cheng R, Robbins A, Wardle J (2011). Parental feeding behaviours and motivations. A qualitative study in mothers of UK pre-schoolers. Appetite.

[30] Egnell M, Crosetto P, d’Almeida T, Kesse-Guyot E, Touvier M, Ruffieux B (2019). Modelling the impact of different front-of-package nutrition labels on mortality from non-communicable chronic disease. Int J Behav Nutr Phys Act.

[31] Boyland EJ, Nolan S, Kelly B, Tudur-Smith C, Jones A, Halford JC (2016). Advertising as a cue to consume: a systematic review and meta-analysis of the effects of acute exposure to unhealthy food and nonalcoholic beverage advertising on intake in children and adults. Am J Clin Nutr.

[32] Mills SDH, Tanner LM, Adams J (2013). Systematic literature review of the effects of food and drink advertising on food and drink-related behaviour, attitudes and beliefs in adult populations. Obes Rev.

[33] Darley WK, Lim JS (2023). Advertising creativity and its effects: a meta-analysis of the moderating role of modality. Mark Lett.

[34] Vukmirovic M (2015). The effects of food advertising on food-related behaviours and perceptions in adults: A review. Food Res Int.

[35] Riedel AS, Weeks CS, Beatson AT (2018). Am I intruding? Developing a conceptualisation of advertising intrusiveness. J Mark Manag.

[36] Fransen M, Verlegh P, Kirmani A, Smit E (2015). A typology of consumer strategies for resisting advertising, and a review of mechanisms for countering them. Int J Advert.

[37] Fourquet-Courbet MP, Courbet D (2009). Analyse de la réception des messages médiatiques Récits rétrospectifs et verbalisations concomitantes. Comm Langages.

