# Supplementary material for: A randomized controlled trial to test the effects of displaying the Nutri-Score in food advertising on consumer perceptions and intentions to purchase and consume
Source: Int J Behav Nutr Phys Act. 2024 Apr 15;21:38. doi: 10.1186/s12966-024-01588-5 (PMC11017538; doi:10.1186/s12966-024-01588-5)
Supplement: Supplementary file 1 — Additional file 1. Material: products, advertisements, and quantity-frequency scales used in the experiment. [file 12966_2024_1588_MOESM1_ESM.docx]

**Additional file 1.** Material: products, advertisements, and quantity-frequency scales used in the experiment.

| **Food category Amount-frequency scale** | **Products** | **Nutri-Score** | **Advertising Examples (see the translation of the advertisements into English below the table)** |
| --- | --- | --- | --- |
| **Cookies**  *Regarding your intention to consume this product, would you say that you intend to consume:*  7. 4 cookie(s) or more/day  6. 1 to 3 cookie(s)/day  5. 4 to 6 cookie(s)/week  4. 1 to 3 cookie(s)/week  3. 1 to 3 cookie(s)/month  2. A few cookies/year  1. Never |  |  |  |
|  | **Brands of the French retailer**  Lemon flavor tile | C | 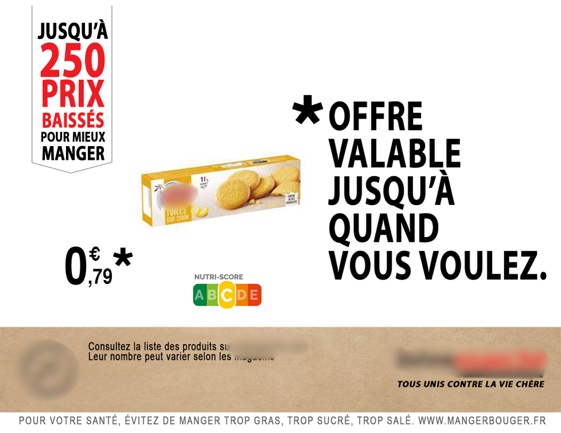 |
|  | Madeleines | D |  |
|  | Speculoos | E |  |
|  |  |  |  |
| **Cereals**  *Regarding your intention to consume this product, would you say that you intend to consume:*  7. 2 or more servings/day  6. 1 serving/day  5. 4 to 6 servings/week  4. 1 to 3 serving(s)/week  3. 1 to 3 serving(s)/month  2. A few servings/year  1. Never |  |  |  |
|  | Oatmeal | A | 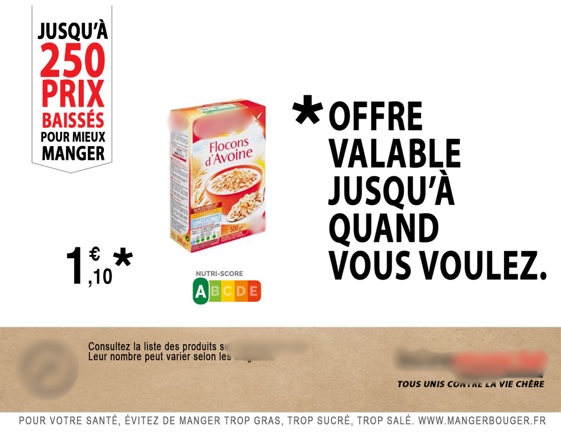 |
|  | Red fruit shape | B |  |
|  | Muesli Crisp 4 nuts | C |  |
|  | Muesli Crisp 3 chocolates | D |  |
|  |  |  |  |
| **Breakfast**  *Regarding your intention to consume this product, would you say that you intend to consume:*  7. 8 slices or more/day  6. 4 to 7 slices/day  5. 1 to 3 slices/day  4. 4 to 6 slices/week  3. 1 to 3 slices/month  2. A few slices in the year  1. Never |  |  |  |
|  | Grand Mie | A | 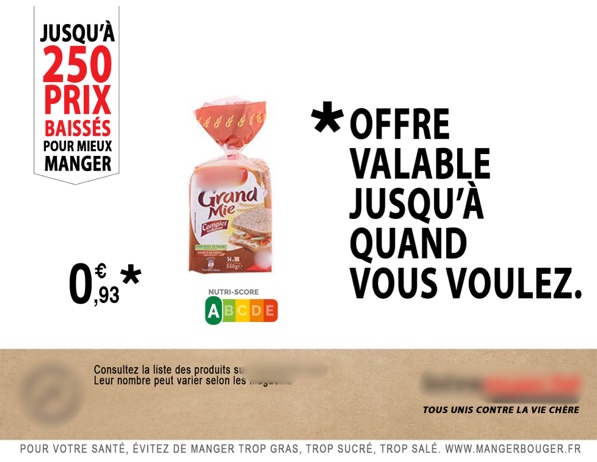 |
|  | Toasted buns | B |  |
|  | Rusks | C |  |
|  | Sliced Brioche | D |  |
|  | 16 chocolate rolls | E |  |
|  |  |  |  |
| **Ready meals**  *Regarding your intention to consume this product, would you say that you intend to consume:*  7. 2 courses and more/day  6. 1 dish/day  5. 4 to 6 dishes/week  4. 1 to 3 dishes/week  3. 1 to 3 dishes/month  2. A few dishes during the year  1. Never |  |  |  |
|  | Basque chicken | A | 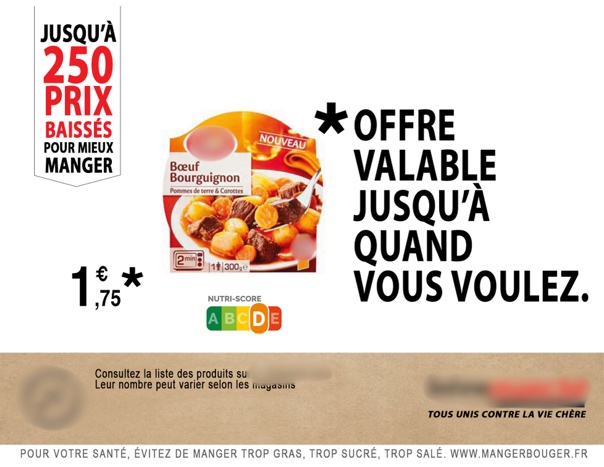 |
|  | Rabbit with 2 mustards | C |  |
|  | Beef bourguignon | E |  |
|  |  |  |  |

| **Salty Snacks**  *Regarding your intention to consume this product, would you say that you intend to consume:*  7. 2 handfuls of aperitif biscuits and more/day  6. 1 handful of aperitif biscuits/day  5. 4 to 6 handfuls of aperitif biscuits/week  4. 1 to 3 handful(s) of crackers/week  3. 1 to 3 handfuls of aperitif biscuits/month  2. A few handfuls of crackers/year  1. Never |  |  |  |
| --- | --- | --- | --- |
|  | Tortilla Chips | C | 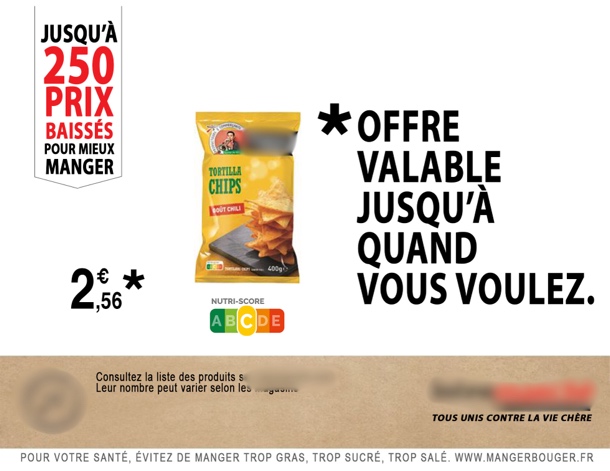 |
|  | Roof tiles | D |  |
|  | crackers | E |  |
|  | chocolate cereal | B |  |
|  | Strawberry cereal | C |  |
|  | Cereals | C |  |
|  | Apples | D |  |
|  | Chocolate bar | E |  |
|  |  |  |  |
| **Beverages**  *Regarding your intention to consume this product, would you say that you intend to consume:*  7. 2 or more glasses/day  6. 1 glass/day  5. 4 to 6 glasses/week  4. 1 to 3 glasses/week  3. 1 to 3 glasses/month  2. A few glasses/year  1. Never |  |  |  |
|  | Water | A | 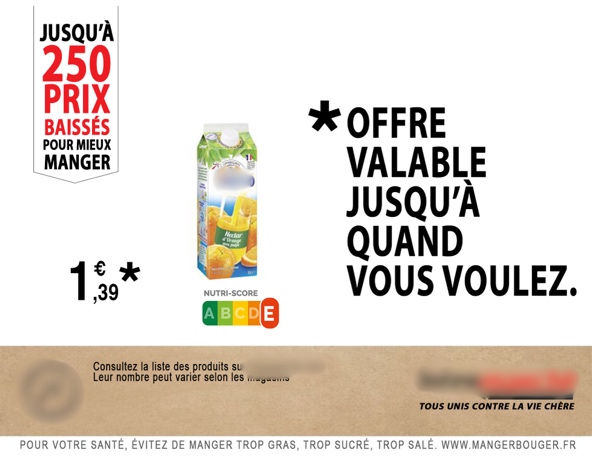 |
|  | Lime Water | B |  |
|  | Pure orange juice | C |  |
|  | Peach Tea | D |  |
|  | Multifruit juice | D |  |
|  | Cola | E |  |
|  | Lemonade | E |  |
|  | orange nectar | E |  |
|  |  |  |  |
| **Cold cuts** |  |  |  |
| *Regarding your intention to consume this product, would you say that you intend to consume:*  7. 2 or more slices/day  6. 1 slice/day  5. 4 to 6 slices/week  4. 1 to 3 slices/week  3. 1 to 3 slices/month  2. A few slices in the year  1. Never | Ham of Paris | C | 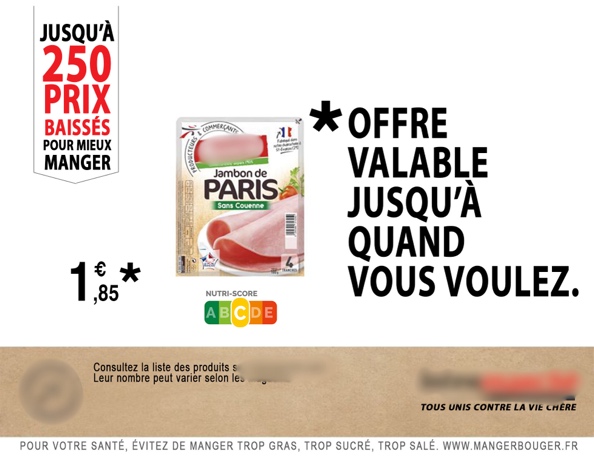 |
|  | Bacon fillet | D |  |
|  | Country pâté | E |  |
|  |  |  |  |
| **Bars**  *Regarding your intention to consume this product, would you say that you intend to consume:*  7. 2 or more bars/day  6. 1 bar/day  5. 4 to 6 bars/week  4. 1 to 3 bars/week  3. 1 to 3 bars/month  2. A few bars/year  1. Never |  |  |  |
|  | Oat bran | A | 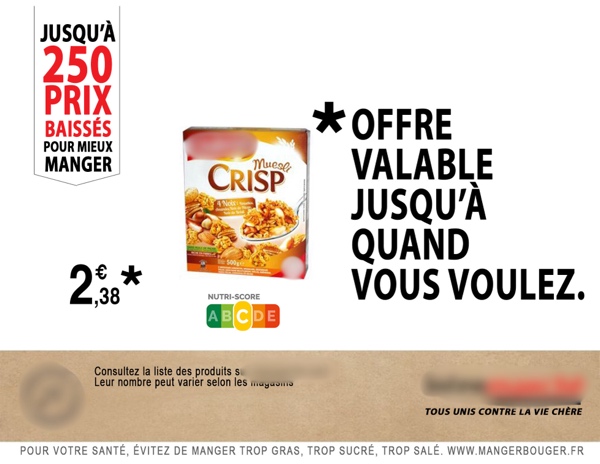 |
|  | chocolate cereal | B |  |
|  | Strawberry cereal | C |  |
|  | Cereals | C |  |
|  | Apples | D |  |
|  | Chocolate bar | E |  |
|  |  |  |  |

| **Desserts**  *Regarding your intention to consume this product, would you say that you intend to consume:*  7. 2 or more jars/day  6. 1 jar/day  5. 4 to 6 jar(s)/week  4. 1 to 3 jar(s)/week  3. 1 to 3 jar(s)/month  2. A few jars/year  1. Never |  |  |  |
| --- | --- | --- | --- |
|  | Applesauce | A | 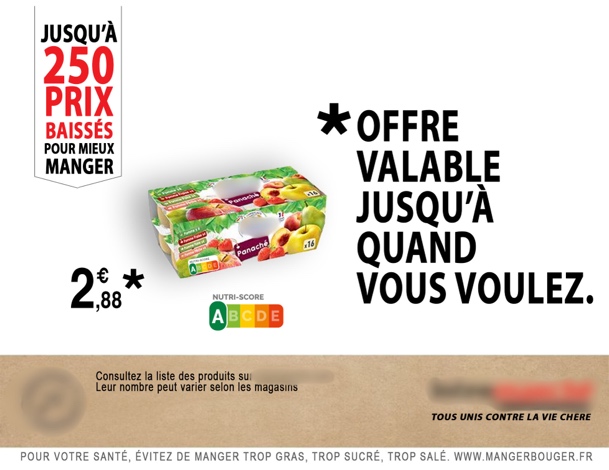 |
|  | Strawberry Yogurt | B |  |
|  | Caramel Creams | C |  |
|  | Crème Brulée | D |  |
|  |  |  |  |
| Notes. Translation of advertising messages into English: *Offer valid until whenever you want - Up to 250 lower prices to eat better. All united against high prices.* | | | |
