# Supplementary material for: A randomized controlled trial to test the effects of displaying the Nutri-Score in food advertising on consumer perceptions and intentions to purchase and consume
Source: Int J Behav Nutr Phys Act. 2024 Apr 15;21:38. doi: 10.1186/s12966-024-01588-5 (PMC11017538; doi:10.1186/s12966-024-01588-5)
Supplement: Supplementary file 2 — Additional file 2. Results: Descriptive statistics (all food categories considered). [file 12966_2024_1588_MOESM2_ESM.docx]

**Additional file 2. Results: Descriptive statistics (all food categories considered)**

|  |  | **Overall Score** | | | | | | | | | |  |
| --- | --- | --- | --- | --- | --- | --- | --- | --- | --- | --- | --- | --- |
|  |  | **NS A** |  | **NS B** |  | **NS C** |  | **NS D** |  | **NS E** |  |  |
|  |  | m |  | m |  | m |  | m |  | m |  |  |
|  |  | (SD) |  | (SD) |  | (SD) |  | (SD) |  | (SD) |  |  |
| Control Condition |  | 3.10 |  | 2.84 |  | 2.63 |  | 2.51 |  | 2.26 |  |  |
|  |  | (1.34) |  | (1.05) |  | (1.00) |  | (0.91) |  | (0.88) |  |  |
| Messages without NS |  | 2.82 |  | 2.70 |  | 2.54 |  | 2.41 |  | 2.19 |  |  |
|  |  | (1.22) |  | (1.02) |  | (1.05) |  | (0.96) |  | (0.86) |  |  |
| Messages with NS |  | 3.65 |  | 3.23 |  | 2.52 |  | 2.09 |  | 1.73 |  |  |
|  |  | (1.35) |  | (1.09) |  | (0.84) |  | (0.80) |  | (0.72) |  |  |

|  |  | **Perceived product nutritional quality** | | | | | | | | | |  | **Perceived healthiness of the product** | | | | | | | | | |
| --- | --- | --- | --- | --- | --- | --- | --- | --- | --- | --- | --- | --- | --- | --- | --- | --- | --- | --- | --- | --- | --- | --- |
|  |  | **NS A** | **n** | **NS B** | **n** | **NS C** | **n** | **NS D** | **n** | **NS E** | **n** |  | **NS A** | **n** | **NS B** | **n** | **NS C** | **n** | **NS D** | **n** | **NS E** | **n** |
|  |  | m |  | m |  | m |  | m |  | m |  |  | m |  | m |  | m |  | m |  | m |  |
|  |  | (SD) |  | (SD) |  | (SD) |  | (SD) |  | (SD) |  |  | (SD) |  | (SD) |  | (SD) |  | (SD) |  | (SD) |  |
| Control Condition |  | 3.97 | 5381 | 3.65 | 5313 | 3.16 | 7866 | 2.93 | 7539 | 2.47 | 6844 |  | 3.96 | 5381 | 3.69 | 5313 | 3.23 | 7866 | 3.01 | 7539 | 2.64 | 6844 |
|  |  | (1.42) |  | (1.22) |  | (1.11) |  | (1.10) |  | (1.06) |  |  | (1.32) |  | (1.11) |  | (1.01) |  | (1.01) |  | (0.98) |  |
| Messages without NS |  | 3.72 | 5408 | 3.55 | 5224 | 3.06 | 7824 | 2.84 | 7449 | 2.44 | 6799 |  | 3.78 | 5408 | 3.60 | 5224 | 3.14 | 7824 | 2.95 | 7449 | 2.63 | 6799 |
|  |  | (1.36) |  | (1.22) |  | (1.11) |  | (1.07) |  | (1.06) |  |  | (1.28) |  | (1.09) |  | (1.01) |  | (0.98) |  | (0.98) |  |
| Messages with NS |  | 5.03 | 5418 | 4.43 | 5228 | 3.18 | 7825 | 2.31 | 7556 | 1.64 | 6746 |  | 4.66 | 5418 | 4.20 | 5228 | 3.19 | 7825 | 2.56 | 7556 | 2.00 | 6746 |
|  |  | (1.51) |  | (1.22) |  | (0.95) |  | (0.87) |  | (0.87) |  |  | (1.35) |  | (1.10) |  | (0.90) |  | (0.88) |  | (0.92) |  |

|  |  | **Affective evaluation of the product (attitude)** | | | | | | | | | |  | **Intention to purchase the product** | | | | | | | | | |
| --- | --- | --- | --- | --- | --- | --- | --- | --- | --- | --- | --- | --- | --- | --- | --- | --- | --- | --- | --- | --- | --- | --- |
|  |  | **NS A** | **n** | **NS B** | **n** | **NS C** | **n** | **NS D** | **n** | **NS E** | **n** |  | **NS A** | **n** | **NS B** | **n** | **NS C** | **n** | **NS D** | **n** | **NS E** | **n** |
|  |  | m |  | m |  | m |  | m |  | m |  |  | m |  | m |  | m |  | m |  | m |  |
|  |  | (SD) |  | (SD) |  | (SD) |  | (SD) |  | (SD) |  |  | (SD) |  | (SD) |  | (SD) |  | (SD) |  | (SD) |  |
| Control Condition |  | 3.59 | 5381 | 3.43 | 5313 | 3.12 | 7866 | 2.98 | 7539 | 2.69 | 6844 |  | 2.27 | 5381 | 2.02 | 5313 | 2.06 | 7866 | 1.99 | 7539 | 1.79 | 6844 |
|  |  | (1.49) |  | (1.30) |  | (1.17) |  | (1.18) |  | (1.19) |  |  | (1.62) |  | (1.41) |  | (1.26) |  | (1.23) |  | (1.15) |  |
| Messages without NS |  | 3.43 | 5408 | 3.34 | 5224 | 3.04 | 7824 | 2.90 | 7449 | 2.67 | 6799 |  | 2.11 | 5408 | 1.95 | 5224 | 2.01 | 7824 | 1.94 | 7449 | 1.76 | 6799 |
|  |  | (1.44) |  | (1.30) |  | (1.17) |  | (1.16) |  | (1.18) |  |  | (1.51) |  | (1.37) |  | (1.25) |  | (1.21) |  | (1.14) |  |
| Messages with NS |  | 4.21 | 5418 | 3.89 | 5228 | 3.01 | 7825 | 2.47 | 7556 | 2.07 | 6746 |  | 2.72 | 5418 | 2.34 | 5228 | 1.96 | 7825 | 1.70 | 7556 | 1.49 | 6746 |
|  |  | (1.56) |  | (1.33) |  | (1.09) |  | (1.08) |  | (1.10) |  |  | (1.84) |  | (1.58) |  | (1.19) |  | (1.06) |  | (0.93) |  |
|  |  | **Intention to personally consume the product** | | | | | | | | | |  | **Intention to give the product to a child** | | | | | | | | | |
|  |  | **NS A** | **n** | **NS B** | **n** | **NS C** | **n** | **NS D** | **n** | **NS E** | **n** |  | **NS A** | **n** | **NS B** | **n** | **NS C** | **n** | **NS D** | **n** | **NS E** | **n** |
|  |  | m |  | m |  | m |  | m |  | m |  |  | m |  | m |  | m |  | m |  | m |  |
|  |  | (SD) |  | (SD) |  | (SD) |  | (SD) |  | (SD) |  |  | (SD) |  | (SD) |  | (SD) |  | (SD) |  | (SD) |  |
| Control Condition |  | 2.04 | 5381 | 1.84 | 5313 | 1.92 | 7866 | 1.85 | 7539 | 1.73 | 6844 |  | 2.55 | 5381 | 2.39 | 5313 | 2.26 | 7866 | 2.13 | 7539 | 2.01 | 6844 |
|  |  | (1.34) |  | (1.09) |  | (0.95) |  | (0.90) |  | (0.87) |  |  | (1.52) |  | (1.23) |  | (1.04) |  | (0.99) |  | (0.95) |  |
| Messages without NS |  | 1.94 | 5408 | 1.79 | 5224 | 1.88 | 7824 | 1.82 | 7449 | 1.72 | 6799 |  | 2.40 | 5408 | 2.26 | 5224 | 2.21 | 7824 | 2.08 | 7449 | 2.00 | 6799 |
|  |  | (1.27) |  | (1.08) |  | (0.95) |  | (0.90) |  | (0.87) |  |  | (1.44) |  | (1.20) |  | (1.03) |  | (0.98) |  | (0.97) |  |
| Messages with NS |  | 2.31 | 5418 | 2.05 | 5228 | 1.86 | 7825 | 1.68 | 7556 | 1.53 | 6746 |  | 2.93 | 5418 | 2.72 | 5228 | 2.20 | 7825 | 1.90 | 7556 | 1.70 | 6746 |
|  |  | (1.46) |  | (1.22) |  | (0.91) |  | (0.81) |  | (0.74) |  |  | (1.59) |  | (1.34) |  | (0.99) |  | (0.90) |  | (0.84) |  |
| *Note:* NS = Nutri-Score (NS A means Nutri-Score A). SD means standard deviation. | | | | | | | | | | | | | | | | | | | | | | |
