# Supplementary material for: A randomized controlled trial to test the effects of displaying the Nutri-Score in food advertising on consumer perceptions and intentions to purchase and consume
Source: Int J Behav Nutr Phys Act. 2024 Apr 15;21:38. doi: 10.1186/s12966-024-01588-5 (PMC11017538; doi:10.1186/s12966-024-01588-5)
Supplement: Supplementary file 3 — Additional file 3. Descriptive statistics: Values of the overall score for 9 food categories according to the “messages with vs. without the Nutri-Score” variable and the nutritional quality of the products (Nutri-Score from A to E). [file 12966_2024_1588_MOESM3_ESM.docx]

**Additional file 3. Descriptive statistics: Values of the overall score for 9 food categories according to the “messages with vs. without the Nutri-Score” variable and the nutritional quality of the products (Nutri-Score from A to E).**

|  |  | **Cookies** | | | | | | | | | |  | | **Cereals** | | | | | | | | | | |
| --- | --- | --- | --- | --- | --- | --- | --- | --- | --- | --- | --- | --- | --- | --- | --- | --- | --- | --- | --- | --- | --- | --- | --- | --- |
|  |  | **NS A** | **n** | **NS B** | **n** | **NS C** | **n** | **NS D** | **n** | **NS E** | **n** | |  | | **NS A** | **n** | **NS B** | **n** | **NS C** | **n** | **NS D** | **n** | **NS E** | **n** |
|  |  | m |  | m |  | m |  | m |  | m |  | |  | | m |  | m |  | m |  | m |  | m |  |
|  |  | (SE) |  | (SE) |  | (SE) |  | (SE) |  | (SE) |  | |  | | (SE) |  | (SE) |  | (SE) |  | (SE) |  | (SE) |  |
| Messages without NS |  |  |  |  |  | 2.65 | 2023 | 2.71 | 1953 | 2.72 | 1996 | |  | | 3.38 | 1502 | 2.35 | 2983 | 2.36 | 1511 |  |  |  |  |
|  |  |  |  |  |  | (0.02) |  | (0.02) |  | (0.02) |  | |  | | 0.03 |  | 0.02 |  | 0.03 |  |  |  |  |  |
| Messages with NS |  |  |  |  |  | 2.75 | 2042 | 2.35 | 1996 | 2.07 | 1962 | |  | | 4.07 | 1489 | 3.00 | 2985 | 2.43 | 1552 |  |  |  |  |
|  |  |  |  |  |  | (0.02) |  | (0.02) |  | (0.02) |  | |  | | 0.03 |  | 0.02 |  | 0.03 |  |  |  |  |  |

|  |  | **Breakfast** | | | | | | | | | |  | | **Ready meals** | | | | | | | | | | |
| --- | --- | --- | --- | --- | --- | --- | --- | --- | --- | --- | --- | --- | --- | --- | --- | --- | --- | --- | --- | --- | --- | --- | --- | --- |
|  |  | **NS A** | **n** | **NS B** | **n** | **NS C** | **n** | **NS D** | **n** | **NS E** | **n** | |  | | **NS A** | **n** | **NS B** | **n** | **NS C** | **n** | **NS D** | **n** | **NS E** | **n** |
|  |  | m |  | m |  | m |  | m |  | m |  | |  | | m |  | m |  | m |  | m |  | m |  |
|  |  | (SE) |  | (SE) |  | (SE) |  | (SE) |  | (SE) |  | |  | | (SE) |  | (SE) |  | (SE) |  | (SE) |  | (SE) |  |
| Messages without NS |  | 2.66 | 1214 | 3.21 | 1195 | 3.02 | 1204 | 2.42 | 1209 | 1.92 | 1232 | |  | | 2.03 | 2010 |  |  | 1.95 | 2039 | 1.90 | 1948 |  |  |
|  |  | (0.03) |  | (0.03) |  | (0.03) |  | (0.03) |  | (0.03) |  | |  | | (0.02) |  |  |  | (0.02) |  | (0.02) |  |  |  |
| Messages with NS |  | 3.45 | 1220 | 3.61 | 1172 | 2.83 | 1212 | 2.17 | 1213 | 1.57 | 1180 | |  | | 2.82 | 2040 |  |  | 2.05 | 1945 | 1.72 | 2031 |  |  |
|  |  | (0.03) |  | (0.03) |  | (0.03) |  | (0.03) |  | (0.03) |  | |  | | (0.02) |  |  |  | (0.02) |  | (0.02) |  |  |  |

|  |  | **Salty Snacks** | | | | | | | | | |  | **Beverages** | | | | | | | | | |
| --- | --- | --- | --- | --- | --- | --- | --- | --- | --- | --- | --- | --- | --- | --- | --- | --- | --- | --- | --- | --- | --- | --- |
|  |  | **NS A** | **n** | **NS B** | **n** | **NS C** | **n** | **NS D** | **n** | **NS E** | **n** |  | **NS A** | **n** | **NS B** | **n** | **NS C** | **n** | **NS D** | **n** | **NS E** | **n** |
|  |  | m |  | m |  | m |  | m |  | m |  |  | m |  | m |  | m |  | m |  | m |  |
|  |  | (SE) |  | (SE) |  | (SE) |  | (SE) |  | (SE) |  |  | (SE) |  | (SE) |  | (SE) |  | (SE) |  | (SE) |  |
| Messages without NS |  |  |  |  |  | 2.05 | 2039 | 1.99 | 1967 | 1.85 | 2004 |  | 4.58 | 545 | 3.15 | 552 | 3.25 | 1800 | 2.87 | 2090 | 2.38 | 2045 |
|  |  |  |  |  |  | (0.02) |  | (0.02) |  | (0.02) |  |  | 0.05 |  | (0.05) |  | (0.03) |  | (0.02) |  | (0.02) |  |
| Messages with NS |  |  |  |  |  | 2.27 | 2018 | 1.95 | 2058 | 1.59 | 1949 |  | 4.89 | 533 | 3.31 | 553 | 3.00 | 1786 | 2.33 | 2074 | 1.75 | 2104 |
|  |  |  |  |  |  | (0.02) |  | (0.02) |  | (0.02) |  |  | 0.05 |  | (0.05) |  | (0.03) |  | (0.02) |  | (0.02) |  |

Note: NS = Nutri-Score (NS A means Nutri-Score A). SE means standard error.

|  |  | **Cold cuts** | | | | | | | | | |  | **Bars** | | | | | | | | | |
| --- | --- | --- | --- | --- | --- | --- | --- | --- | --- | --- | --- | --- | --- | --- | --- | --- | --- | --- | --- | --- | --- | --- |
|  |  | **NS A** | **n** | **NS B** | **n** | **NS C** | **n** | **NS D** | **n** | **NS E** | **n** |  | **NS A** | **n** | **NS B** | **n** | **NS C** | **n** | **NS D** | **n** | **NS E** | **n** |
|  |  | m |  | m |  | m |  | m |  | m |  |  | m |  | m |  | m |  | m |  | m |  |
|  |  | (SE) |  | (SE) |  | (SE) |  | (SE) |  | (SE) |  |  | (SE) |  | (SE) |  | (SE) |  | (SE) |  | (SE) |  |
| Messages without NS |  |  |  |  |  | 2.86 | 2035 | 2.38 | 1979 | 2.03 | 2007 |  | 3.11 |  | 3.11 |  | 2.62 |  | 2.65 |  | 2.04 |  |
|  |  |  |  |  |  | (0.02) |  | (0.02) |  | (0.02) |  |  | (0.04) | 585 | (0.04) | 531 | (0.03) | 1061 | (0.04) | 545 | (0.04) | 557 |
| Messages with NS |  |  |  |  |  | 2.76 | 1997 | 2.11 | 2063 | 1.66 | 1939 |  | 3.65 | 552 | 3.47 | 555 | 2.59 | 1111 | 2.24 | 526 | 1.86 | 562 |
|  |  |  |  |  |  | (0.02) |  | (0.02) |  | (0.02) |  |  | (0.04) |  | (0.04) |  | (0.03) |  | (0.04) |  | (0.04) |  |
| \|  \|  \|  \| \| \| \| \| \| \| \| \| \|  \| \| --- \| --- \| --- \| --- \| --- \| --- \| --- \| --- \| --- \| --- \| --- \| --- \| --- \| \|  \|  \| **Desserts** \| \| \| \| \| \| \| \| \| \|  \| \|  \|  \| **NS A** \| **n** \| **NS B** \| **n** \| **NS C** \| **n** \| **NS D** \| **n** \| **NS E** \| **n** \|  \| \|  \|  \| m \|  \| m \|  \| m \|  \| m \|  \| m \|  \|  \| \|  \|  \| (SE) \|  \| (SE) \|  \| (SE) \|  \| (SE) \|  \| (SE) \|  \|  \| \| Messages without NS \|  \| 3.13 \| 1565 \| 2.99 \| 1436 \| 2.39 \| 1509 \| 2.53 \| 1517 \|  \|  \|  \| \|  \|  \| (0.03) \|  \| (0.03) \|  \| (0.03) \|  \| (0.03) \|  \|  \|  \|  \| \| Messages with NS \|  \| 4.02 \| 1472 \| 3.48 \| 1491 \| 2.51 \| 1510 \| 2.12 \| 1518 \|  \|  \|  \| \|  \|  \| (0.03) \|  \| (0.03) \|  \| (0.03) \|  \| (0.03) \|  \|  \|  \|  \|   Note: NS = Nutri-Score (NS A means Nutri-Score A). SE means standard error. | | | | | | | | | | | | | | | | | | | | | | |
